# Reallocation of time spent on sedentary behavior by time spent on physical activity reduces dynapenia in older adults: a prospective cohort study

**DOI:** 10.1590/1516-3180.2022.0188.R2.20092022

**Published:** 2022-12-19

**Authors:** Rizia Rocha Silva, Lucas Lima Galvão, Giovana Silva Martins, Joilson Meneguci, Jair Sindra Virtuoso-Júnior, Douglas de Assis Teles Santos, Sheilla Tribess

**Affiliations:** I MSc. Student, Postgraduate Program in Physical Education, Universidade Federal do Triângulo Mineiro (UFTM), Uberaba (MG), Brazil.; II MSc. Student, Postgraduate Program in Physical Education, Universidade Federal do Triângulo Mineiro (UFTM), Uberaba (MG), Brazil.; III MSc. Student, Postgraduate Program in Physical Education, Universidade Federal do Triângulo Mineiro (UFTM), Uberaba (MG), Brazil.; IV PhD. Professor, Postgraduate Program in Physical Education, Universidade Federal do Triângulo Mineiro (UFTM), Uberaba (MG), Brazil.; V PhD. Professor, Postgraduate Program in Physical Education, Universidade Federal do Triângulo Mineiro (UFTM), Uberaba (MG), Brazil.; VI PhD. Professor, Faculty of Physical Education, Universidade do Estado da Bahia (UNEB), Teixeira de Freitas (BA), Brazil.; VII PhD. Professor, Postgraduate Program in Physical Education, Universidade Federal do Triângulo Mineiro (UFTM), Uberaba (MG), Brazil.

**Keywords:** Aged, Muscle strength, Epidemiology, Aging, Exercise, Sitting time, Grip strength, Physical activities, Sedentary time

## Abstract

**BACKGROUND:**

Dynapenia is characterized by mobility limitations in the older population when combined with aggravating behavioral factors that can increase the risk of morbidity and mortality.

**OBJECTIVE:**

To investigate the hypothetical effects of reallocation of time spent on sedentary behavior (SB), moderate-to-vigorous physical activity (MVPA), and sleep on dynapenia in older adults.

**DESIGN AND SETTING:**

A prospective cohort study using exploratory surveys in Alcobaça City, Bahia State, Brazil.

**METHODS:**

In total, 176 older adults (≥ 60 years) of both sexes participated in this study. Dynapenia was assessed using the handgrip strength test with cutoff points of < 27 kg for men and < 16 kg for women. MVPA and SB were assessed using the International Physical Activity Questionnaire, and sleep was assessed using the Pittsburgh Sleep Quality Index.

**RESULTS:**

Effects on reallocation were found for the shortest times, such as 10 minutes (odds ratio (OR) 0.92; 95% confidence interval (CI): 0.85–0.99); substituting MVPA with SB increased the chances of dynapenia by 58.0% (95% CI: 1.01–2.49). Analyzing the substitution of 60 minutes/day of SB with 60 minutes/day of MVPA revealed a protective effect, with a lower OR for dynapenia of 37.0% (OR 0.63; 95% CI: 0.40–0.99). The reallocation of sleep time did not significantly reduce dynapenia.

**CONCLUSIONS:**

Substituting the time spent sitting with the same amount of time spent on MVPA can reduce dynapenia, and a longer reallocation time confers greater health benefits in older adults.

## INTRODUCTION

Aging is commonly accompanied by a significant reduction in muscle performance, since skeletal muscle mass and strength are affected by this process.^1^ The age-related decline in muscle strength is termed dynapenia. This condition exposes older adults to a greater risk of mobility limitations.^2^ It is directly influenced by behavioral factors such as the level of physical activity (PA), exposure to sedentary behavior (SB), and quality and duration of sleep.^3^

Moderate-to-vigorous physical activity (MVPA) is an established component of healthy aging and can improve the health and longevity of the population.^4^ Insufficient levels of physical activity are prevalent worldwide; in older adults, this prevalence reportedly ranges from 4.9% (Sweden)^5^ and 29.0% (Portugal)^5^ to 33% in Brazil.^6^ PA levels among older adults remain below the minimum 150 to 300 minutes per week recommended by the World Health Organization.^7^ These low levels induce several deleterious muscle adaptations, including reductions in muscle volume, power, and strength, which are aggravating factors for older adults.^8^

Concomitantly, advancing age has been associated with high SB,^3^ with an estimated sedentary time of older adults of 9.4 hours per day, ranging from 8.5 to 10.7 hours per day, according to a systematic review of 22 studies.^9^ Consequently, SB is independently associated with reduced muscle strength, which contributes to reducing the functionality and autonomy of older adults.^10^

Therefore, exposure to dynapenia may play a role in the relationship between PA, MVPA, and SB. Establishing and quantifying the associations between such variables is thus a priority for informing potential lifestyle guidelines and interventions, ultimately mitigating poor health outcomes.^11^

Regarding sleep, its relationship with aging and strength and its close association with the development of adverse health conditions have been described.^12^ A study found that low handgrip strength was independently associated with poor sleep quality in middle-aged and older adults.^13^

Although the association between SB, PA, and sleep has been investigated in the literature,^14,15^ studies examining the relationship between dynapenia and SB, PA, and sleep, especially their effects when assessing the reallocation of the exposure time of older individuals to these activities, are lacking. Therefore, investigating sleep hour time, MVPA, and SB in relation to dynapenia is relevant; an isotemporal substitution modeling shows the ability not only to control the effect between activities but also the effect of substitutions of time spent, reducing the heterogeneity of associations, thus facilitating public health recommendations.^16^ We hypothesized that the hypothetical reallocation of time in MVPA by SB would increase the odds of dynapenia.

## OBJECTIVE

To investigate the hypothetical effects of the reallocation of time spent on SB, MVPA, and sleep on dynapenia in older adults.

## METHODS

### Study design

This was a prospective and observational cohort study, part of the Longitudinal Study of Elderly Health in Alcobaça (ELSIA, as per its Portuguese acronym) conducted between 2015 and 2020 in the municipality of Alcobaça, located in the extreme south of state of Bahia, Brazil. It comprised 743 older adults aged 60 years and over who lived in urban areas and were registered in the Family Health Strategy (FHS). This program comprises a care model to access public health, aiming to promote the integration of social security services with the public health services of states and municipalities.^17^

### Participants

For the survey, individuals registered in the FHS of the Health System of the Brazilian government, conducted in Alcobaça, were selected. Alcobaça has 743 older adults enrolled in the FHS; 54 of whom refused to participate in the survey, 58 were excluded because they did not meet the inclusion criteria, and 158 could not be located, resulting in a final sample of 473 individuals.^18^ The exclusion criteria were severe cognitive impairment according to the Mini-Mental State Examination (MMSE), adapted for the Brazilian population,^19^ severe difficulty in visual and hearing acuity, use of wheelchairs, severe sequelae of stroke with localized loss of strength, or terminal illness. For home visits, the researchers used data provided by the Municipal Health Department of Alcobaça as a reference. Contact was made with the older adults through home visits, informing them of the objectives, and requesting their participation in the research voluntarily.^20^

**Figure 1.**
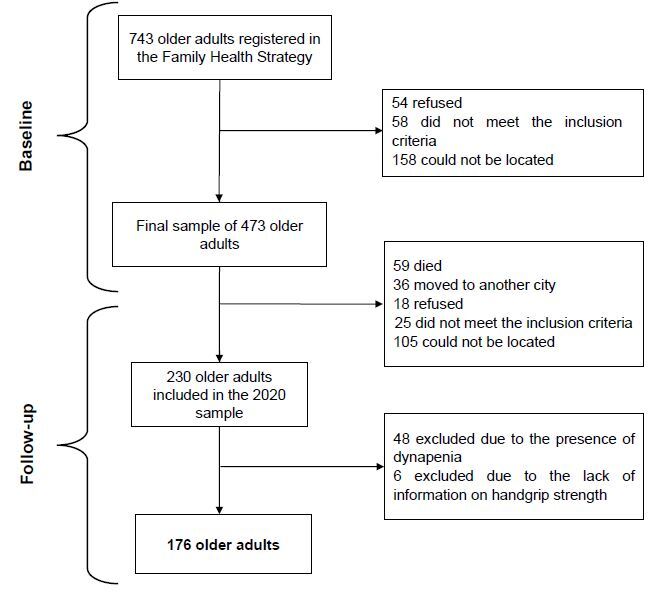
Longitudinal Study of Elderly Health in Alcobaça, 2015–2020, Sample flowchart.

In February 2020, 249 participants were excluded due to a lack of information (59 due to death, 36 due to relocation to another city, 18 due to refusal to participate, 25 due to not meeting the inclusion criteria, and 105 due to not being locatable); 48 were excluded because they already had dynapenia at the beginning of the study, and 6 were excluded due to a lack of information on handgrip strength, resulting in a final study population of 176 individuals (Figure 1).

### Ethical consideration

This study complied with the procedures and protocols of the Declaration of Helsinki and was approved by the Research Ethics Committee of the Universidade Federal do Triângulo Mineiro (no. 966.983/2015; date: February 25, 2015) and the Universidade do Estado da Bahia (no. 3.471.114/2020; date: July 26, 2019). Participation was voluntary and all participants provided informed consent.

### Dynapenia

Dynapenia was assessed using the handgrip strength test with a Jamar portable hydraulic dynamometer (SAEHAN, SH5001, Korea). The participants were instructed to remain standing, with their elbows extended, then press the handle of the dynamometer with the highest force possible and hold it for 6 seconds. The recovery time between attempts was 1 minute. Three measurements were obtained in kilograms/force (kgf). The highest value of attempts for the dominant hand (self-reported by the subject) was used in the analysis.^21^

Dynapenia was classified as < 27 kgf for men and < 16 kgf for women, according to the criteria of Dodds et al.^22^

### Physical activity and sedentary behavior

PA and SB were assessed using the long form of the International Physical Activity Questionnaire (IPAQ), validated for Brazilian older adults.^23,24^

PA was determined based on activities with MVPA for at least 10 continuous minutes during one day of the week. To characterize older individuals, a cutoff point of 150 minutes/week of MVPA was used (≥ 150 minutes/week = sufficiently active and < 150 minutes/week = insufficiently active),^7^ and for the reallocation analysis, the time of MVPA was used continuously.

SB was determined by the time spent sitting during one day in the week and one day on the weekend. The total time spent sitting (minutes/day) was determined based on the weighted arithmetic mean [(time sitting on a weekday × 5 + time sitting on a weekend Day × 2)/7].^25^ The 50^th^ percentile of sitting time, corresponding to 391.78 minutes/day, was used as the cutoff point to characterize older individuals with high SB (≥ 50^th^ percentile). For isotemporal analyses, total continuous values were used.

### Sleep

The time spent on nocturnal sleep was measured by the question, “During the past month, how many hours did you sleep at night?,” from the Pittsburgh Sleep Quality Index,^26^ translated and validated for Brazilian Portuguese.^27^ It refers to the amount of sleep an individual has per night. Continuous values expressed as minutes per day (minutes/day) were considered for the construction of the isotemporal substitution models.

### Covariables

Data on socioeconomic and general health variables were collected using a structured questionnaire. The variables consisted of sex (male and female), age group (60–69, 70–79, and ≥ 80 years), marital status (with a partner and without a partner), occupation (paid work and without paid work), income (value in financial unit BRL converted to American dollars U$) and schooling (years of study), polypharmacy (0 to 4 medicines ≥ 5 medicines), Basic Activities of daily living (BADL) (score) was assessed by using the Katz Index,^28^ number of diseases (amount), smoking (yes or no) self-reported by the participant. The body mass index (BMI) was calculated as body mass/height² (kg/m²). The waist-hip ratio (WHR) was determined by measuring the circumference in centimeters (cm) and was defined as waist to umbilical scar and hip at the largest circumference of the gluteal bone through the ratio of one measure to the other (cm waist/hip cm).^29^

### Data analysis

Epidata software, version 3.1b, was used to prepare the database, and the analyses were performed using SPSS software (version 23.0; SPSS, Inc. Chicago, Illinois, United States). The Kolmogorov–Smirnov test was used to test the normality of the data.

Descriptive statistics were used to identify the sample, including the distribution of absolute and relative frequencies, medians, means, standard deviations (SDs), and interquartile ranges. The difference between groups with and without dynapenia was measured using the Mann–Whitney *U* test. For the association between the covariables and dynapenia, inferential statistics were used (Pearson’s chi-square test).

To determine the hypothetical effects of the reallocation of time spent on sleep, SB, and PA on dynapenia, the isotemporal substitution approach was used.^30^ Isotemporal substitution analyses were performed using logistic regression, with an estimate of odds ratio (OR) and 95% confidence interval (CI). The effects of substituting the times of 10, 20, 30, 40, 50, and 60 minutes spent on sleep, SB, and MVPA for the presence of dynapenia were also checked. The models were adjusted for sex, basic activities of daily living scores, income, smoking, number of diseases, polypharmacy, schooling, body mass index, and waist-hip ratio. A significance level of 5% was used.

## RESULTS

This study included 176 older adults of both sexes, with a median age of 66.0 years. The incidence of dynapenia during the follow-up period was 17% (n = 30). Table 1 displays the characteristics of the participants and their associations with the covariables at baseline, according to the incidence of dynapenia at follow-up.

**Table 1.** Characteristics of participants and associations according to the presence and absence of dynapenia. Alcobaça-BA, Brazil, 2020

Variables	Total n (%)	Dynapenia		P
		Absence	Presence	
		n (%)	n (%)	
**Sex**				
Male	63 (35.8)	50 (79.4)	13 (20.6)	0.404
Female	113 (64.2)	96 (85.0)	17 (15.0)	
**Age group**				
60–69 years	120 (68.2)	106 (88.3)	14 (11.7)	**0.003**
70–79 years	46 (26.1)	35 (76.1)	11 (23.9)	
≥ 80 years	10 (5.7)	5 (50.0)	5 (50.0)	
**Marital status**				
Without partner	87 (49.4)	69 (79.3)	18 (20.7)	0.233
With partner	89 (50.6)	77 (86.5)	12 (13.5)	
**Occupation**				
Employed	48 (27.3)	42 (87.5)	6 (12.5)	0.376
Unemployed	128 (72.7)	104 (81.3)	24 (18.3)	
**Polypharmacy**				
0–4 medicines	148 (84.1)	128 (86.5)	20 (13.5)	**0.011**
≥ 5 medicines	28 (15.9)	18 (64.3)	10 (35.7)	
**Smoking**				
No	160 (90.9)	133 (75.6)	27 (16.9)	0.739
Yes	16 (9.1)	13 (81.3)	3 (18.8)	
**Level of physical activity**				
≥ 150 minutes/week	114 (64.8)	98 (86.0)	16 (14.0)	0.207
< 150 minutes/week	62 (35.2)	48 (77.4)	14 (22.6)	
**Sedentary lifestyle**				
< 535 minutes/day	142 (80.7)	120 (84.5)	22 (15.5)	0.309
≥ 535 minutes/day	34 (19.3)	26 (76.5)	8 (23.5)	
	**Median (IQR)**	**Median (IQR)**	**Median (IQR)**	**P**
**Income (Dollars)**	322.81 (326.96)	326.96 (284,54)	322.81 (326.96)	0.179
**Number of Diseases**	3.00 (4.00)	3.00 (4.00)	3.50 (4.00)	0.379
**Schooling (years)**	4.00 (6.00)	4.52 (5.00)	3.50 (6.00)	0.828
**BMI (kg/m²)**	27.01 (6.93)	27.03 (6.54)	26.78 (7.61)	0.196
**WHR (cm)**	0.98 (0.10)	0.98 (0.10)	0.99 (0.13)	0.406

Data are expressed as absolute and relative frequencies for categorical variables and as medians and interquartile ranges for quantitative variables. IQR = interquartile range; BMI = body mass index; WHR = waist-to-hip ratio.

The mean times of the measured variables included in the hypothetical isotemporal substitution model were a mean of 64 minutes/day (SD 76.57; IRQ 73.21) for MVPA, a mean of 413.94 minutes/day (SD 149.48; IRQ 173.04) for sedentary behavior and a mean of 414.00 minutes/day (SD 98.36; IRQ 120.00) for sleep.

In the isotemporal substitution analyses (Table 2), it was observed that the substitution of MVPA time for time spent on SB resulted in a higher OR of dynapenia at all tested times of 10, 20, 30, 40, 50, and 60 minutes among the surveyed older individuals (P < 0.05).

**Table 2.** Isotemporal substitution model of the association among sleep time reallocation, sedentary behavior, and moderate to vigorous physical activity in the risk of dynapenia in older adults. Alcobaça-BA, Brazil, 2020

Substitution Models	Dynapenia		
	OR (95% CI)	OR (95% CI)	OR (95% CI)
	MVPA	SB	Sleep
**10 minutes**			
MVPA Substitution	-	1.08 (1.01–1.16)^*^	1.05 (0.95–1.14)
SB Substitution	0.92 (0.85–0.99)^*^	-	0.97 (0.92–1.02)
Sleep Substitution	0.95 (0.87–1.04)	1.02 (0.97–1.08)	-
**20 minutes**			
MVPA Substitution	-	1.16 (1.01–1.35)^*^	1.10 (0.92–1.31)
SB Substitution	0.85 (0.73–0.99)^*^	-	0.94 (0.84–1.05)
Sleep Substitution	0.90 (0.75–1.08)	1.05 (0.95–1.17)	-
**30 minutes**			
MVPA Substitution	-	1.26 (1.01–1.58)^*^	1.15 (0.88–1.51)
SB Substitution	0.79 (0.63–0.99)^*^	-	0.91 (0.78–1.07)
Sleep Substitution	0.86 (0.66–1.13)	1.09 (0.92–1.28)	-
**40 minutes**			
MVPA Substitution	-	1.36 (1.01–1.83)^*^	1.21 (0.84–1.74)
SB Substitution	0.73 (0.54–0.99)^*^	-	0,89 (0.71–1.10)
Sleep Substitution	0.82 (0.57–1.18)	1.12 (0.90–1.39)	-
**50 minutes**			
MVPA Substitution	-	1.47 (1.01–2.14)^*^	1.27 (0.81–2.00)
SB Substitution	0.68 (0.46–0.99)^*^	-	0.86 (0.66–1.13)
Sleep Substitution	0.78 (0.50–1.23)	1.15 (0.88–1.51)	-
**60 minutes**			
MVPA Substitution	-	1.58 (1.01–2.49)^*^	1.33 (0.77–2.29)
SB Substitution	0.63 (0.40–0.99)^*^	-	0.84 (0.61–1.16)
Sleep Substitution	0.74 (0.43–1.28)	1.18 (0.86–1.64)	-

CI = confidence interval; OR = odds ratio; MVPA = moderate to vigorous physical activity; SB = sedentary behavior. Adjusted for sex, basic activities of daily living score, income, smoking, number of diseases, polypharmacy, years of study, body mass index, and waist-hip ratio; ^*^P < 0.005*.*

The reduction in SB and increase in MVPA were shown to have a protective role, where the longer the substitution time, the greater the protective effect. Substituting short times, such as 10 minutes/day of SB, with 10 minutes/day of MVPA was associated with an 8% reduction in dynapenia. In comparison, at the maximum time of 60 minutes/day, reallocation was associated with a 37% reduction in the development of dynapenia (95% CI: 0.40–0.99). Substitutions of sleep time with SB and MVPA times did not result in significant differences.

## DISCUSSION

The main findings show that reallocations of SB by MVPA at all times tested reduced the chances of developing dynapenia. The inverse mode also occurs where the reallocation of time in MVPA by SB is a risk factor for the conservation of muscle strength in older adults.

Recent investigations have shown the possible effect of physical activity on muscle strength.^31,11^ Consistent with these studies, the results of the current study reinforce this positive association, showing PA as a protective factor for reducing muscle strength in the aging process. Cooper et al.,^32^ with a sample of more than 66,000 English citizens aged ≥ 60 years, identified a linear and positively associated behavior of handgrip strength and PA, the older adults whose handgrip strength increased spent more minutes per day on MVPA.

Despite its health benefits, PA levels among older adults remain below the recommended 150 minutes/week.^33^ It has been shown that even at low levels, small changes in the inactive profile can improve and maintain the health of older adults.^3^ These results reinforce that changes in small amounts of time (10 and 20 minutes/day) in the increase of PA showed benefits by significantly reducing the chances of developing dynapenia.

Conversely, SB contributes to an unhealthy lifestyle^34^ associated with declines in performance and muscle strength in older adults.^1^ Accordingly, the results of the current study highlight the risks of time increments in SB for dynapenia from the short times of 10 to 30 minutes per day, in addition to the fact that the reallocation of an additional hour (60 minutes daily) of SB, there was a 58% increase of dynapenia, corroborating the results reported by Gianoudis et al.^35^ for each additional hour.

This factor has been assessed by sedentary activity; the study by Hammer and Stamatakis^10^ addressed the daily time spent on TV and internet use and its inverse association with muscle strength, highlighting that older adults who watched TV for ≥ 6 hours per day had less handgrip strength than older individuals who watched TV for < 2 hours per day.

In older adults, sleep and muscle strength vary according to the aging process. As modifiable parameters, they can interact and influence each other.^36^ The results of the current study did not show significant changes in the reallocations of sleep time by SB or MVPA, which can be explained by the mean sleep rate of the population, which was of the recommended regular amount (~7 hours). However, recent investigations have identified strong evidence between the quality and amount of sleep and muscle strength.^36,37^ Pourmotabbed et al. showed that both short (< 6 hours) and long (> 8 hours) periods of sleep could lead to an increase in the risk of sarcopenia (decline in muscle mass, strength, and performance).^37^

In the isotemporal substitution model, no studies reported on dynapenia as an outcome; however, with sarcopenia and its components, Sánchez-Sánchez et al.^38^ found that the reallocation of 60 minutes/day of MVPA by time spent on SB was associated with a reduction in the risk of sarcopenia (OR = 0.522; 95% CI: 0.367–0.726). Furthermore, when its components were assessed separately, reallocation was also associated with higher handgrip strength values (β = 0.888; 95% CI: 0.145–1.631).

MVPA is an important predictor for the maintenance of muscle physiology,^12^ especially in aging, contributing to the increase of systemic inflammation, improving its oxidative power, and decreasing the loss of motor units, thus helping to conserve muscle strength.^1^ On the other hand, the systems directly involving SB and dynapenia remain unclear; however, physiological processes explain that staying sedentary can influence systemic inflammation, which contributes to the infiltration of adipocytes into muscle tissue,^39^ reducing the contractile capacity of the skeletal muscle that entails, among other outcomes, decreased muscle power and strength,^35^ thus revealing similar paths between SB and dynapenia.

The use of isotemporal substitution modeling demonstrates a valuable avenue for the development of research within the epidemiological area owing to its ability to interdependently identify activities of different intensities, making more realistic assumptions that an increase in a behavior will be accompanied by a decrease in the equal duration of the others while the total time in all behaviors is kept constant.^40^ These findings may be important in preparing specific recommendations for PA and SB in older adults. This can be useful for primary health and health professionals on how to use discretionary time in a way that is beneficial to health in daily practice.

With the need for future studies that complement our results, monitoring the high exposure to SB already present in the population can influence the development of dynapenia, even if PA levels remain above the recommended parameters.

Among the limitations of this study is the isotemporal substitution method, which is hypothetically applied, and the lack of estimating the change in behavior via a direct assessment. Moreover, we implemented an instrument that indirectly assesses PA and SB, which does not estimate mild intensity, which is considered important for the composition of the day in 24 hours. Nevertheless, the strengths of the study should be highlighted, such as its representative sample, the follow-up having been performed by the same assessors throughout the study period, in addition to the measurement of muscle strength with the hydraulic dynamometer, considered the gold standard for large populations, its originality, and its configuration in a longitudinal design that no other studies have utilized.

## CONCLUSIONS

Substitution of the time spent on MVPA with the same amount in SB is associated with an increased risk of dynapenia. The opposite also occurs; longer time spent on MVPA correlates with greater benefits, drastically reducing the risk of developing dynapenia, thus directly reflecting on the reduction of the limiting impacts of the decline in muscle strength.
